# Supplementary material for: The Evolution and Characterization of the RNA Interference Pathways in Lophotrochozoa
Source: Genome Biol Evol. 2024 May 7;16(5):evae098. doi: 10.1093/gbe/evae098 (PMC11114477; doi:10.1093/gbe/evae098)
Supplement: evae098_Supplementary_Data [file evae098_supplementary_data.zip › Supplementary_materials.zip!/selected_miRNAs/NoGe_selected_miRNAs/mirdeep_pdf_files/NMRB01000991.1_Notospermus_geniculatus_strain_Ushimado_scaffold991,_whole_genome_shotgun_sequence_23697.pdf]

Provisional ID	:	NMRB01000991.1_Notospermus_geniculatus_strain_Ushimado_scaffold991,_whole_genome_shotgun_sequence_23697
Score total	:	482.2
Score for star read(s)	:	3.9
Score for read counts	:	477.3
Score for mfe	:	1.5
Score for randfold	:	0
Score for cons. seed	:	-0.6
Total read count	:	948
Mature read count	:	921
Loop read count	:	0
Star read count	:	27

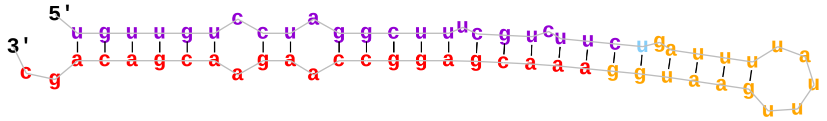

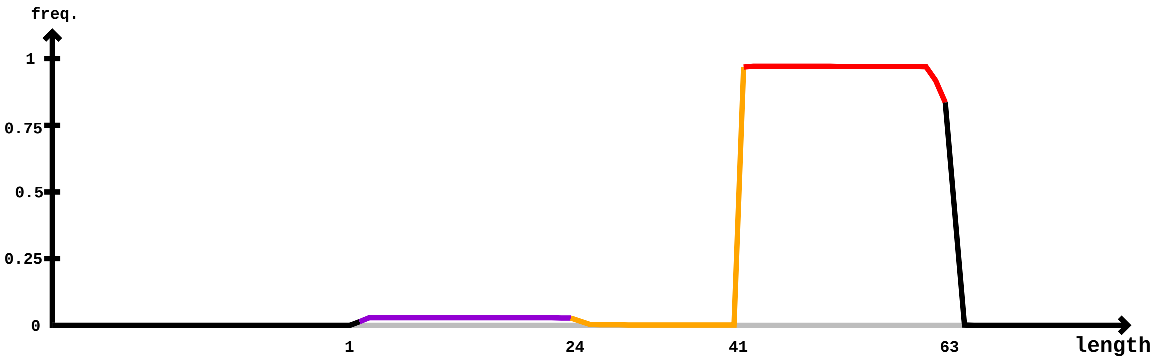

Star Mature

```
5'- cauccucuggauacaacaagccaguacgacuguuguccuaggcuuucgucuucugauuuuauuugaauggaaacgaggccaagaacgacagcugcacuggcauacgucau  -3'   obs
    cauccucuggauacaacaagccaguacgacuguuguccuaggcuuucgucuucugauuuuauuugaauggaaacgaggccaagaacgacagcugcacuggcauacgucau         exp
    .((((...)))).......((((((.((.(((((((.((.(((((.(((.((((.((((.....)))))))))))))))).)).))))))).)).)))))).........     reads    mm      sample
    ......................................................................aaacgaggccaagaacgacaC...................     1        1       Fm2
    ......................................................................aaacgaggccaagaacgacagU..................     52       1       Fm2
    ......................................................................aaacgaggccaagaacgacaCc..................     9        1       Fm2
    ......................................................................aaacgaggccaagaacgacagUu.................     9        1       Fm2

    ..............................uguuguccuaggcuuucgucuuc.........................................................     8        0       Fm1
    ...............................guuguccuaggcuuucgucG...........................................................     1        1       Fm1
    ...............................guuguccuaggcuuucgucuuc.........................................................     6        0       Fm1
    ...............................guuguccuaggcuuucgucuucu........................................................     2        0       Fm1
    ...............................guuguccuaggcuuucgCcuucu........................................................     1        1       Fm1
    ......................................................................aaacgaggccaagaacgacag...................     6        0       Fm1
    ......................................................................aaacgaggccaagaacgacaC...................     1        1       Fm1
    ......................................................................aaacgaggccaagaacgacaCc..................     2        1       Fm1
    ......................................................................aaacgaggccaagaacgacagc..................     60       0       Fm1
    ......................................................................aaacgaggccaagaacgacagU..................     99       1       Fm1
    ......................................................................aaacgaggccaagaacgacagA..................     8        1       Fm1
    ......................................................................aaacgaggccaagaacgacagcA.................     8        1       Fm1
    ......................................................................aaacgaggccaagaacgacagUu.................     3        1       Fm1
    .......................................................................aacgaggccaagaacgacagc..................     1        0       Fm1

    ..............................uguuguccuaggcuuucgucuuc.........................................................     5        0       Mm2
    ...............................guuguccuaggcuuucgucuuc.........................................................     1        0       Mm2
    ...............................guuguccuaggcuuucgucuucuU.......................................................     1        1       Mm2
    .....................................................ugauuuuauuugaauggaaacgaggcc..............................     1        0       Mm2
    ......................................................................aaacgaggccaagaacgaca....................     5        0       Mm2
    ......................................................................aaacgaggccaagaacgacag...................     3        0       Mm2
    ......................................................................aaacgaggccaagaacgacaU...................     1        1       Mm2
    ......................................................................aaacgaggccaagaacgacaC...................     1        1       Mm2
    ......................................................................aaacgaggccaagaacgacagA..................     4        1       Mm2
    ......................................................................aaacgaggccaagaacgacagc..................     34       0       Mm2
    ......................................................................aaacgaggccaagaacgacaCc..................     6        1       Mm2
    ......................................................................aaacgaggccaagaacgUcagc..................     1        1       Mm2
    ......................................................................aaacgaggccaagaacgacagU..................     108      1       Mm2
```

Star Mature

```
cauccucuggauacaacaagccaguacgacuguuguccuaggcuuucgucuucugauuuuauuugaauggaaacgaggccaagaacgacagcugcacuggcauacgucau
......................................................................aaacgaggccaagaacgacaCcu.................   3    1   Mm2
......................................................................aaacgaggccaagaacgacagUu.................   3    1   Mm2
......................................................................aaacgaggccaagaacgacagcA.................   3    1   Mm2

......................................................................aaacgaggccaagaacgacaC...................   2    1   Mm1
......................................................................aaacgaggccaagaacgacagA..................   16   1   Mm1
......................................................................aaacgaggccaagaacgacaCc..................   10   1   Mm1
......................................................................aaacgaggccaagaacgacagc..................   5    0   Mm1
......................................................................aGacgaggccaagaacgacagc..................   1    1   Mm1
......................................................................aaacgaggccaagaacgacagU..................   7    1   Mm1

......................................................................aaacgaggccaagaacgac.....................   1    0   Fm3
......................................................................aaacgaggccaagaacCaca....................   1    1   Fm3
......................................................................aaacgaggccaagaacgaca....................   38   0   Fm3
......................................................................aaacgaggccaagaacCacag...................   1    1   Fm3
......................................................................aaacgaggccaagaacgacaC...................   9    1   Fm3
......................................................................aaacgaggccaagaacgacag...................   41   0   Fm3
......................................................................aaacgaggccaagaacgCcagc..................   1    1   Fm3
......................................................................aaacgaggccaagaacgacagc..................   65   0   Fm3
......................................................................aaacgaggcUaagaacgacagc..................   1    1   Fm3
......................................................................aaacgaggccaagaacgacagA..................   15   1   Fm3
......................................................................aaacgaggccaagaacgGcagc..................   1    1   Fm3
......................................................................aaacgaggccaagaacgacagU..................   141  1   Fm3
......................................................................aaacgaggccaagaacgacaCc..................   9    1   Fm3
......................................................................aaacgaggccaagaacgacagcu.................   1    0   Fm3
......................................................................aaacgaggccaagaacgacagUu.................   3    1   Fm3
......................................................................aaacgaggccaagaacgacagcA.................   3    1   Fm3
......................................................................aaacgaggccaagaacgacagcuU................   1    1   Fm3
.......................................................................aacgaggccaagaacgacagU..................   1    1   Fm3

...............................guuguccuaggcuuucgucuucu........................................................   1    0   Mm3
...............................guuguccuaggcuuucgucuucugauu....................................................   1    0   Mm3
......................................................................aaacgaggccaagaacgaca....................   5    0   Mm3
......................................................................aaacgaggccaagaacgCcag...................   1    1   Mm3
......................................................................aaacgaggccaagaacgacag...................   11   0   Mm3
......................................................................aaacgaggccaagaacgacagc..................   50   0   Mm3
......................................................................aaacgaggccaagaacgacagA..................   2    1   Mm3
......................................................................aaacgaggccaagaacgacaCc..................   4    1   Mm3
......................................................................aaacgaggccaagaacgacagU..................   40   1   Mm3
......................................................................aaacgaggccaagaacgacagUu.................   1    1   Mm3
......................................................................aaacgaggccaagaacgacagcA.................   1    1   Mm3
.......................................................................aacgaggccaagaacgacagc...................   1    0   Mm3
```
